# Protective Effect of Minocycline Against Ketamine-Induced Injury in Neural Stem Cell: Involvement of PI3K/Akt and Gsk-3 Beta Pathway

**DOI:** 10.3389/fnmol.2016.00135

**Published:** 2016-12-20

**Authors:** Yang Lu, Shan Lei, Ning Wang, Pan Lu, Weisong Li, Juan Zheng, Praveen K. Giri, Haixia Lu, Xinlin Chen, Zhiyi Zuo, Yong Liu, Pengbo Zhang

**Affiliations:** ^1^Department of Anesthesiology, The Second Affiliated Hospital of Xi’an Jiaotong UniversityXi’an, China; ^2^Institute of Neurobiology, National Key Academic Subject of Physiology of Xi’an Jiaotong UniversityXi’an, China; ^3^Department of Anesthesiology, University of VirginiaCharlottesville, VA, USA

**Keywords:** minocycline, ketamine, neural stem cells, neuroprotective effect, Akt, Gsk-3β, caspase-3

## Abstract

It has been suggested that ketamine cause injury during developing brain. Minocycline (MC) could prevent neuronal cell death through the activation of cell survival signals and the inhibition of apoptotic signals in models of neurodegenerative diseases. Here we investigated the protective effect of MC against ketamine-induced injury in neural stem cells (NSCs) from neonatal rat. Ketamine (100 μM/L) significantly inhibited NSC proliferation, promoted their differentiation into astrocytes and suppressed neuronal differentiation of NSCs. Moreover, the apoptotic level was increased following ketamine exposure. MC pretreatment greatly enhanced cell viability, decreased caspase-3-like activity, even reversed the differentiation changes caused by ketamine. To elucidate a possible mechanism of MC’ neuroprotective effect, we investigated the phosphatidylinositol 3-kinase (PI3K) pathway using LY294002, a specific PI3K inhibitor. Immunoblotting revealed that MC enhanced the phosphorylation/activation of Akt and phosphorylation/inactivation of glycogen synthase kinase-3beta (Gsk-3β). Our results suggest that PI3K/Akt and Gsk-3β pathway are involved in the neuroprotective effect of MC.

## Introduction

Ketamine, a noncompetitive blocker of N-methyl-D-aspartate (NMDA) receptor, is usually used in children as anesthetics. Numerous studies have indicated that ketamine induce both neuron and neural stem cell (NSC) injury in the developing brains in recent years, which calls for caution with its use in neonatal and pediatric anesthesia (Ikonomidou et al., 1999; Hayashi et al., 2002; Young et al., 2005; Slikker et al., 2007; Yan et al., 2014). Thus, development of adjunctive neuroprotective measures that prevent or ameliorate neurotoxicity of ketamine is highly warranted.

As a second generation tetracycline, minocycline (MC) showed neuroprotective effects after both focal and global cerebral ischemia (Yrjänheikki et al., 1998, 1999). It has also been reported that the disease progression of amyotrophic lateral sclerosis and Huntington disease would be delayed with MC treatment in mouse (Chen et al., 2000; Wang C. X. et al., 2003; Wang X. et al., 2003). MC also protects neurons against excitotoxic damage and nigral cell loss (Du et al., 2001; He et al., 2001; Tikka and Koistinaho, 2001; Tikka et al., 2001; Wu et al., 2002). And evidences indicated that, independently of its antimicrobial effect, MC has anti-neuroinflammatory properties via inhibiting microglia activation in the models of TBI-induced focal injury (Bye et al., 2007; Homsi et al., 2010) and matter damage after excitotoxic striatal injury (Guimarães et al., 2010). Some other mechanisms have also been suggested to explain the neuroprotective properties of MC, such as inhibition of caspase-1, caspase-3 (Chen et al., 2000), inducible nitric oxide synthase (iNOS) expression (Amin et al., 1996; Lin et al., 2001), the mitochondrial permeability transition pore (Du et al., 2001), mitochondrial swelling (He et al., 2001), cytochrome c release (Zhu et al., 2002) and inhibition of p38 MAP kinase (Lin et al., 2001). However, whether MC protects NSCs from ketamine-induced injury remains to be elucidated.

Akt is downstream target of phosphatidylinositol 3-kinase (PI3K). The PI3K/Akt cascade has been reported to inhibit apoptosis (Chalecka-Franaszek and Chuang, 1999) and promote cell survival through insulin and growth factors (Franke et al., 1995; Du et al., 1997). After phosphoinositide-dependent protein kinase (PDK) phosphorylating Akt-1, glycogen synthase kinase (Gsk) 3β, a serine/threonine kinase, is inhibited (Grimes and Jope, 2001; Hur and Zhou, 2010). Studies have demonstrated that Gsk-3β play an important role in fundamental functions of cell, such as cell cycle, cytoskeletal integrity, apoptosis, transcription factors expression and formation of neurofibrillary tangles (Cross et al., 1995; Hetman et al., 2000; Grimes and Jope, 2001; Kandimalla et al., 2016). For example, Gsk-3β regulates neurogenesis, neuronal polarization and axon growth in the developing brain (Hur and Zhou, 2010). These findings implied that PI3K kinase/Akt signaling pathway is related to the survival of NSCs.

Given that MC improved the neurological outcome of cerebral ischemia and some other neurodegenerative diseases, we hypothesized that MC protects NSCs from ketamine-induced impairment. Further, we analyzed the intracellular signal transduction cascades involved in our study which showed that the Akt/Gsk-3β pathway is related to MC protection against the damage caused by ketamine.

## Materials and Methods

### Cell Culture

New-born Sprague-Dawley rats were obtained from Laboratory Animal Centre of Xi’an Jiaotong University School of Medicine. This experiment was in accordance with the recommendations of the National Institutes of Health Guide for the Care and Use of Laboratory Animals (NIH Publications No. 80-23) and the protocols were authorized by Committee on Animal Care of Xi’an Jiaotong University. The animal procedures were designed to minimize the number of animals required, and appropriate steps were taken to avoid unjustified animal procedures. This study was not involved in any vulnerable populations, for example minors, persons with disabilities or endangered animal species. Rat primary NSC cultures were prepared from the dissected cortex of Sprague-Dawley rats in 18–19 days of gestation under sterile condition. Briefly, the forebrain portion dissected and placed in ice-cold Hank’s solution (without Mg^2+^ and Ca^2+^, Gibco, Carlsbad, CA, USA). The cells were then dissociated by mechanical agitation through a fire-polished Pasteur pipette. After centrifugation, the isolated cells were resuspended in free-serum DMEM/F12 medium (Gibco, Carlsbad, CA, USA) which was supplemented with 2% B27 (Gibco, Carlsbad, CA, USA), 20 ng/ml EGF (Gibco, Carlsbad, CA, USA), 20 ng/ml bFGF (Gibco, Carlsbad, CA, USA) and 100 U/ml penicillin and phytomycin. A half of culture medium was replaced every 3 days. According to the neurosphere method developed by Reynolds and Weiss (Reynolds and Weiss, 1992), cells were incubated for 7 days to form enough neurospheres. Then the cells were passaged at a density of 2 × 10^5^ cells/ml followed by collection and dissociation. The medium was changed every 3 days. After the second passage, nestin-positive cells were enriched in neurospheres with minimal presence of β-tubulin III or glial fibrillary acidic protein (GFAP)-positive cells, which indicated the characteristic of the NSC preparation.

### Measurement of Cell Viability

After passage, 5 × 10^3^ cells were seeded each well in 96-well plates, then Cell Counting Kit-8 (CCK-8, Beyotime Institute of Biotechnology, shanghai, China) was used to detect the cell viability by adding 10 μl CCK-8 solution per well. At the wavelength of 450 nm on a microplate reader, the Optical density (OD) value was measured 4 h later, and the value was corrected by subtracting the absorbance of control wells that did not contain cells. Data were collected from at least three independent experiments.

### 5-Bromo-2′-deoxyuridine (BrdU) Incorporation Assay

After passage, the cells were seeded on cover slips which processed by 100 μg/mL poly-L-lysine and incubated with the proliferation marker 5-Bromo-2′-deoxyuridine (BrdU; 10 μmol/L, Sigma-Aldrich Inc. St. Louis, MO, USA) for 4 h. Before staining with 4′,6-diamidino-2-phenylindole (DAPI) and BrdU antibody (Sigma-Aldrich Inc. St. Louis, MO, USA) cells were fixed by 4% paraformaldehyde. Immunoreactive cells were visualized by fluorescence microscopy (Olympus, BX51, Japan) and a total of 5–7 randomly selected fields were captured. The minimal number of cells for counting was 200 in each condition, and data were collected from at least three independent experiments.

### Measurement of Cell Differentiation

After passage, the cells were seeded on cover slips which processed by 100 μg/mL poly-L-lysine and incubated with differentiating medium which contains 100 × N2 supplement, 100 × B27 supplement and 1% fetal bovine serum (FBS, Gibco, Carlsbad, CA, USA) in DMEM/F12 (without b-FGF). At the end of 7 day cultures, cells were harvested for differentiation analysis. A total of 5–7 randomly selected images were taken in each well, and the number of cells expressing β-tubulin III, GFAP tested by β-tubulin III antibody (Sigma-Aldrich Inc. St. Louis, MO, USA) and GFAP antibody (Sigma-Aldrich Inc. St. Louis, MO, USA) were counted (at least 200 cells each test case), and data were collected from at least three independent experiments.

### Western Blot Analysis

Following exposure to ketamine and/or MC, cells were washed with ice-cold D-hanks’ solution three times and then lysed with lysis buffer containing protease and phosphatase inhibitors. The cell lysates were placed on ice for 15 min and centrifuged at 14,000 rpm for 15 min at 4°C. Protein concentrations in the resulting lysates were determined with a BCA protein assay kit, bovine serum albumin (BSA) was used as a standard. Equal amount of the resulting cell lysate was resolved by sodium dodecyl sulfate-polyacrylamide gel and the separated proteins were transferred to polyvinylidene fluoride membranes. The membranes were incubated with blocking buffer for 1 h at room temperature and then incubated for 16 h at 4°C with primary antibodies (Cell Signal Technology Inc, Beverly, MA, USA) raised to β-tubulin III, GFAP, caspase-3, phospho-Akt, Akt, phosphor-Gsk-3β, Gsk-3β and β-actin. The signals were detected using goat anti-rabbit or anti-mouse horseradish secondary antibody followed by enhanced chemiluminescence (ECL). After exposing to X-ray films, blots from at least three independent tests were quantified as OD values according to their controls.

### Statistical Analysis

For statistical analysis, differences between animals were evaluated through one-way analysis of variance (ANOVA) and *post hoc* Duncon’s test. Data which expressed as the mean ± SEM were analyzed by SPSS for Windows version 18.0 and Prism 5. Statistical significance was set at *P* < 0.05.

## Results

### Culture and Identification of NSCs

**Figure 1:**
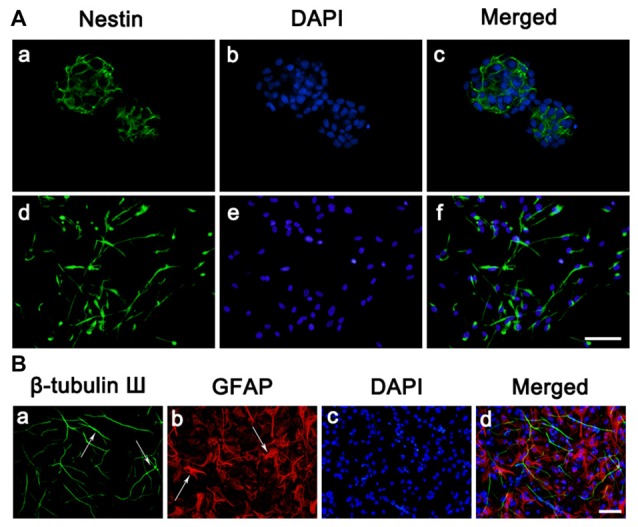
**Culture and identification of neural stem cells (NSCs). (A)** Cell morphology and nestin expressions. **(a–c)** Immunostaining of the Nestin (green) in neurosphere before seeded on substrates. **(d–f)** Immunostaining of the Nestin (green) in neurosphere after seeded on PLL. Cell nuclei was stained with 4′,6-diamidino-2-phenylindole (DAPI; blue). **(B)** Representative immunofluorescence images of the cultures at day 7, as one characteristic of NSCs, the cells differentiated into neurons and astrocytes. **(a)** Immunostaining of the β-tubulin III (green) of the cultures. **(b)** Immunostaining of the glial fibrillary acidic protein (GFAP; red) of the cultures. **(c)** Cell nuclei was stained with DAPI (blue). **(d)** Merge picture of **(a–c)**. Scale bar = 25 μm.

To begin this experiment, neocortical tissues of E18-19 Sprague-Dawley rats were dissected for NSC culture. The suspended growth of neurospheres was notably observed 3 days after seeding. As we can see cells expressing Nestin (Figure 1A), a marker for NSCs, reached to 50%–60% of total cells in a neurosphere, which was consistent with the previous study. In the adherent culture system, NSCs also expressed Nestin (Figure 1A) after seeded on PLL. It is known that neurons, astrocytes and oligodendrocytes could be generated from NSCs by differentiation. To determine this feature of NSCs, cells were immunostained against β-tubulinIII, a specific maker for neuron (green) and GFAP, a specific maker for astrocytes (red; Figure 1B) after 7 days of differentiation. Overall, most of cells in this experiment were NSCs which characterized by the proliferation potential and the capability to differentiate into multiple cell types.

### Minocycline Elicited a Concentration-Dependent Viability Increase in NSCs Exposed to Ketamine

**Figure 2:**
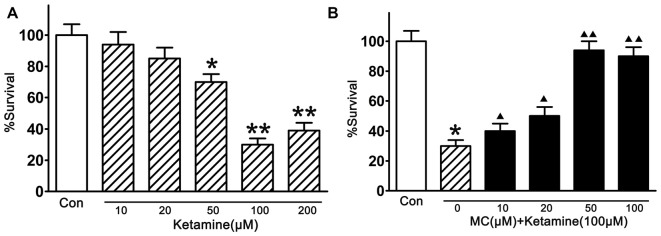
**Inhibition by minocycline (MC) on ketamine-induced injury. (A)** Ketamine induced a dose-dependent damage. NSCs were exposed to 10–200 μM/L ketamine for 24 h, then cell viability was measured by Cell Counting Kit-8 (CCK-8) assay. **(B)** MC prevented ketamine-induced injury dose-dependently. NSCs were exposed to different concentrations of 10–100 μM/L MC for 30 min before the addition of 100 μM/L ketamine. Cell viability was measured by CCK-8 assay at 24 h after ketamine addition. Data, as expressed percentage of control (Con), are the mean ± SEM of four separate experiments (*n* = 6 for each group). *p < 0.05 and **p < 0.01 vs. control; ^▲^*P* < 0.05 and ^▲▲^*P* < 0.01 vs. ketamine.

In this study, the injury of ketamine in cortical NSCs were first tested by CCK-8 (Figure 2A). Compared with vehicle controls, exposed to ketamine (from 50 μM/L to 200 μM/L, Sigma-Aldrich Inc. St. Louis, MO, USA) for 24 h significantly decreased cell viability, and 100 μM/L ketamine resulted in the minimal survival of 33.3% of NSCs. Pre-treatment of NSCs with MC (10, 20, 50, 100, 200 μM/L, Sigma-Aldrich Inc. St. Louis, MO, USA) for 30 min before ketamine exposure, the survival rate of NSCs was 39.5 ± 8%, 50.6 ± 6%, 92.8 ± 5% and 87.7 ± 8%, respectively (Figure 2B), indicating dose-dependent neuroprotective effect of MC on NSCs. It is worth to notice that there was no significant difference in the cell number between groups with different doses of MC. The maximal rescue occurred at a concentration of 50 μM/L MC. Therefore, NSCs were co-treated with 100 μM/L ketamine and 50 μM/L MC for the further study.

### Minocycline Elicited a Time-Dependent Viability Increase in NSCs Exposed to Ketamine and Phosphatidylinositol 3-Kinases (PI3K) Pathway was Involved

**Figure 3:**
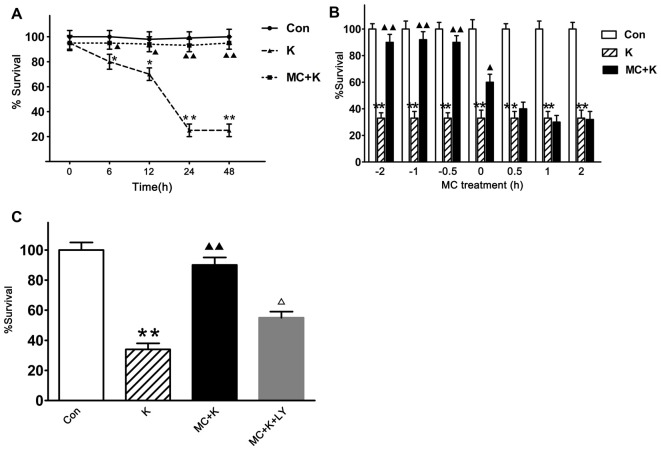
**MC elicits a time-dependent protective effect against ketamine-induced injury, but this protection was inhibited by specific phosphatidylinositol 3-kinase (PI3K) inhibitor LY294002.** Cell survival was measured at 24 h after ketamine exposure by CCK-8 assay. **(A)** Viability comparison of normal NSCs with those exposed to 100 μM/L ketamine alone or exposed to 100 μM/L ketamine plus 50 μM/L MC for 6–48 h. **(B)** NSCs were exposed to 50 μM/L MC at −2 h, −1 h or −0.5h before 100 μM/L ketamine and 0 h, 0.5 h, 1 h or 2 h after ketamine. **(C)** Pre-incubation with LY294002 (20 μM/L) resulted in the elimination of the protective effect induced by 50 μM/L MC. Data are the mean ± SEM of three independent experiments (*n* = 6 for each group). *p < 0.05 and **p < 0.01 vs. control; ^▲^*P* < 0.05 and ^▲▲^*P* < 0.01 vs. ketamine. ^Δ^*P* < 0.05 vs. ketamine plus MC.

To evaluate the neuroprotection of MC persistently, the cell viability at 0 h, 6 h, 12 h, 24 h and 48 h were tested and we found that 100 μM/L ketamine caused a time-dependent viability decrease of NSCs but 50 μM/L MC exhibited protective effect lasting from 6 h to 48 h after ketamine exposure, besides the viability of NSCs in the control group did not change at the above time points (Figure 3A). To establish the temporal profile of MC’s effect, NSCs were exposed to 50 μM/L MC for 0.5 h, 1 h, 2 h before ketamine was added. In order to examine the acute effect of MC, it was also added to NSC culture at 0 h, 0.5 h, 1 h, 2 h after ketamine exposure. Cell viability was examined 24 h after ketamine insult. The result indicated that MC induced a time-dependent viability increase in NSCs, but considering the experimental operability and clinical practical, NSCs were pretreated with MC 30 min before ketamine exposure for the further study. It is worth noting that even when added at the same time with ketamine, NSC viability increased gently but significantly by MC treatment (Figure 3B). However, if adding at 30 min after ketamine exposure, MC did not display any protective effect against NSCs exposed to ketamine.

To investigate whether the protective effect of MC had connection with PI3K signaling pathway, LY294002 was used 120 min before ketamine exposure. The survival rate of NSCs was 92.7 ± 7% after preconditioned with MC. However, with LY294002 (20 μM/L), NSC viability was approximately 57.2 ± 6%, indicating that MC lost its protection in the presence of LY294002 (Figure 3C).

### Minocycline Changed the Effect of Ketamine on NSC Proliferation

**Figure 4:**
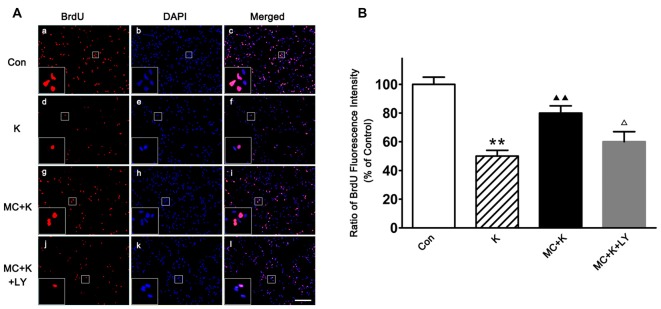
**Effect of MC on NSC proliferation by 5-Bromo-2′-deoxyuridine (BrdU) incorporation assay. (A)** Representative immunofluorescence images of BrdU positive cells in the four groups. Cells were stained for BrdU (red) and DAPI for the nucleus (blue). Scale bar = 50 μm. **(B)** Quantitative analysis of BrdU staining in the experimental groups. Dates are the mean ± SEM of three independent experiments (*n* = 6 for each group); **P < 0.01 vs. control; ^▲▲^*P* < 0.01 vs. ketamine; ^Δ^*P* < 0.05 vs. ketamine plus MC.

NSC proliferation was determined by BrdU incorporation staining, as an analog of thymidine, BrdU can be incorporated into the newly synthesized DNA of replicating cells during the S phase of the cell cycle. After ketamine exposure (100 μM/L; 24 h), BrdU positive cells were remarkably decreased compared to the control. On the contrary, 50 μM/L MC barely restored the proliferation rate to normal level. However, in the group pretreated with LY294002 (20 μM/L), the proliferation rate of NSCs was only 62.2 ± 7% of control group, indicating that PI3K pathway maybe involved in the protective effect induced by MC (Figures 4A,B).

### Minocycline Alleviated the Effect of Ketamine on NSC Differentiation

**Figure 5:**
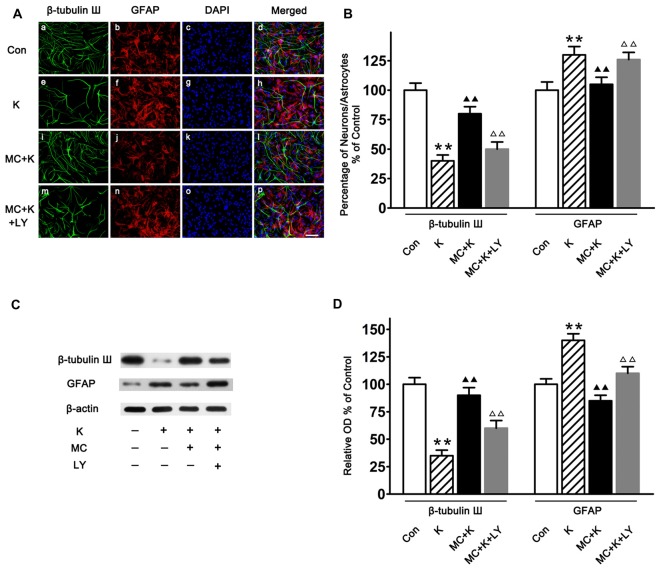
**NSC differentiation on day 7 of culture. (A)** Representative immunofluorescence images of NSCs differentiated neurons and astrocytes in the four groups. Cells were stained for β-tubulin III (green), GFAP (red) and DAPI for the nucleus (blue). Scale bar = 25 μm. **(B)** Quantitative analysis of β-tubulin III and GFAP staining in the experimental groups. Dates are the mean ± SEM of three independent experiments (*n* = 6 for each group); **P < 0.01 vs. control; ^▲▲^*P* < 0.01 vs. ketamine; ^ΔΔ^*P* < 0.01 vs. ketamine plus MC. **(C)** Representative images of β-tubulin III and GFAP expression by western blot in the four groups. **(D)** Quantitative analysis of β-tubulin III and GFAP expressions normalized to β-actin. Data were expressed as the ratio to optical density (OD) values of the corresponding controls and showed as the mean ± SEM of three independent experiments (*n* = 6 for each group); **P < 0.01 vs. control; ^▲▲^*P* < 0.01 vs. ketamine; ^ΔΔ^*P* < 0.01 vs. ketamine plus MC.

Both immunostaining (Figures 5A,B) and western blot (Figures 5C,D) demonstrated that the cultured cells can differentiate into different type of cells, including astrocytes and neurons. Furthermore, it was shown that after ketamine treatment, the percentage of neuron was decreased, and MC increased the differentiation of NSCs towards neuron (Figures 5B,D). On the contrary, MC preconditioning strongly inhibited NSC differentiation towards astrocytes which was increased by ketamine exposure (Figures 5B,D). The effect of MC on NSC differentiation was reversed by LY294002, indicating that PI3K pathway maybe involved in the protective effect induced by MC (Figure 5).

### Minocycline Alleviated Ketamine-Induced Apoptosis by Activating the PI3K Signaling Pathway

**Figure 6:**
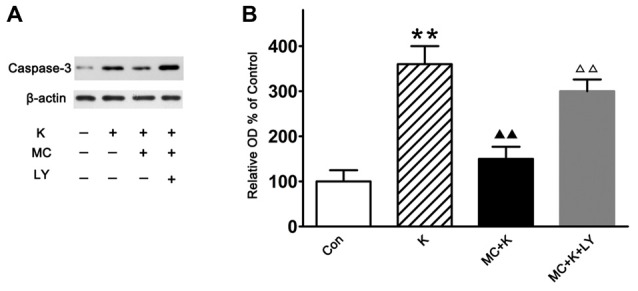
**Western blot analysis of the caspase-3 expression. (A)** Representative images of the caspase-3 and β-actin expression by western blot in the four groups. **(B)** Quantitative analysis of caspase-3 expressions normalized to β-actin. Data were expressed as the ratio to OD values of the corresponding controls and showed as the mean ± SEM of three independent experiments (*n* = 6 for each group); **P < 0.01 vs. control; ^▲▲^*P* < 0.01 vs. ketamine; ^ΔΔ^*P* < 0.01 vs. ketamine plus MC.

Caspase-3 is often associated with apoptosis. In this study, experiments were carried out by western blot analysis to detect the difference of caspase-3 expression between three groups. Ketamine exposure produced an increase in the expression of caspase-3 compared with the control. Pretreatment with 50 μM/L MC decreased the caspase-3 expression after ketamine exposure. However, co-treatment with 20 μM/L LY294002, reversed the protective effect of MC (Figures 6A,B).

### Minocycline Reversed the Inhibitory Effect of Ketamine to PI3K/Akt Pathway

**Figure 7:**
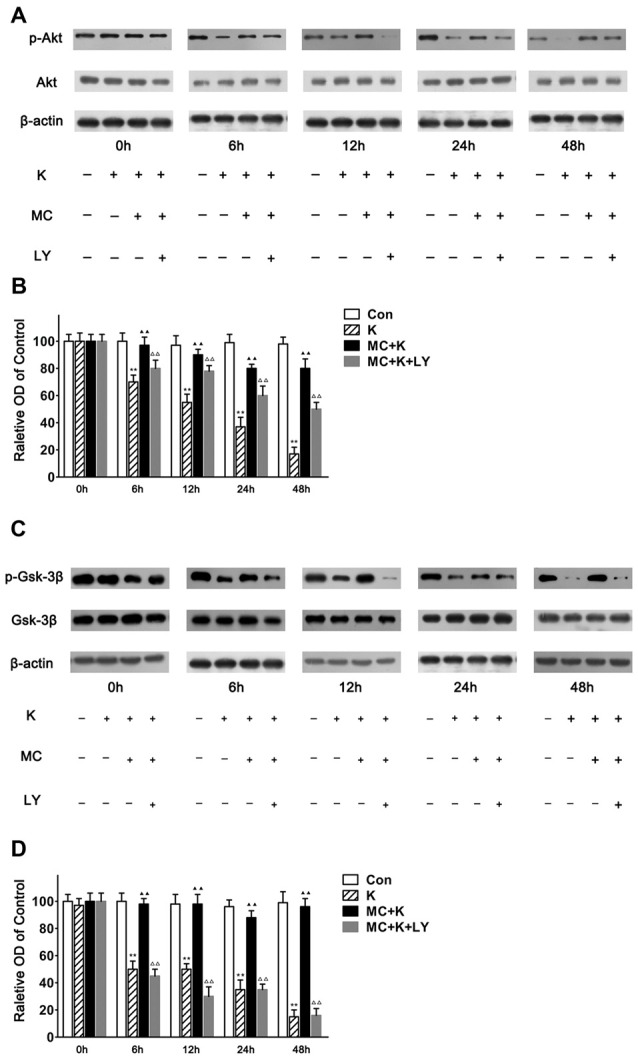
**MC reverses the inhibitory effect of ketamine by PI3K/Akt pathway. (A,B)** MC time-dependently prevented ketamine-evoked decrease of p-Akt. **(A)** Representative images of p-Akt, Akt and β-actin expression by western blot in the four groups. **(B)** Quantitative analysis of p-Akt, Akt and β-actin expressions normalized to β-actin. **(C,D)** MC time-dependently prevented ketamine-evoked decrease of p-Gsk-3β. **(C)** Representative images of p-Gsk-3β, glycogen synthase kinase-3beta (Gsk-3β) and β-actin expression by western blot in the four groups. **(D)** Quantitative analysis of p-Gsk-3β, Gsk-3β and β-actin expressions normalized to β-actin. Data were expressed as the ratio to OD values of the corresponding controls and showed as the mean ± SEM of three independent experiments (*n* = 6 for each group); **P < 0.01 vs. control; ^▲▲^*P* < 0.01 vs. ketamine; ^ΔΔ^*P* < 0.01 vs. ketamine plus MC.

As number of previous researches indicated that the neuroprotection of MC was associated with the phosphorylation of Akt and considering that LY294002 almost completely abolished the protection of MC to NSC injury induced by ketamine, we examined the expression level of Akt/PKB, a downstream effector of PI3K which had been demonstrated to promote cell survival. In this study NSCs exposed to ketamine for 6 h lead to decreased p-Akt levels significantly, which last for 48 h in a time-dependent manner. Pretreatment with MC reversed the effect of ketamine in p-Akt levels in NSCs. Moreover, 20 μM/L LY294002 weakened the protective effect of MC for at least 48 h. There was no significant difference in total amount of Akt in our observation (Figures 7A,B).

It is known that decreased p-Akt induces lower phosphorylation of Gsk-3β to p-Gsk-3β. Therefore, the level of phosphorylated Gsk-3β was analyzed at 0 h, 6 h, 12 h, 24 h and 48 h followed by ketamine treatment. In this study NSCs exposed to ketamine for 6 h leads to decreased p-Gsk-3β level significantly, which lasts for 48 h in a time-dependent manner. Pretreament with MC reversed the effect of ketamine on p-Gsk-3β levels in NSCs. Moreover, 20 μM/L LY294002 weakened the protective effect of MC for at least 48 h. No alteration in the total amount of Gsk-3β was observed (Figures 7C,D). Our results indicated that Gsk-3β, as the crucial substrate of Akt, was involved in the protective effect of MC against ketamine insult.

## Discussion

Ketamine has been reported to induce injury in the developing brain (Liu et al., 2011), whereas how ketamine affect the neurogenesis in this period is not very clear. Given the importance of NSCs in neurogenesis, we investigated the effect of ketamine on proliferation and differentiation of NSCs from embryonic day 18–19 rat *in vitro*. We also observed whether MC can prevent ketamine-induced injury and the possible intracellular signaling pathway. The key findings are that MC attenuated ketamine-induced NSC injury by inhibiting Gsk-3β activity and maintaining the PI3K/Akt pathway activation *in vitro*.

NSCs are self-renewal and multipotential cells that can generate neurons and glia. It plays an important role in neurogenesis and gliogenesis in the development of the central nervous system. In humans, the brain growth spurt (BGS) proceeds from the third trimester until approximately 2 years after birth. In rats, this period corresponds to the first 21 postnatal days (PNDs). We investigated the effect of ketamine on NSCs from embryonic day 18–19 rats, which is equivalent to human BGS.

The potential toxic effect of different concentration of ketamine on cultured NSCs were examined first, it was observed that ketamine (10 and 20 μM/L) failed to alter the NSC viability. Ketamine at the concentration of 50, 100 and 200 μM/L significantly inhibited the NSC viability through PI3K/Akt/Gsk-3β signaling pathway *in vitro*. Studies have shown that plasma concentration of ketamine in different patients who were given 2.0–2.2 mg/kg of it by intravenous injection would reach a peak at 108.4 μM vs. a valley at 37.8 μM 1 min later (Domino et al., 1982). So further investigation definitely need to be done to explore the effect of ketamine on the brain development at clinical relevant doses.

Neurogenesis is characterized by three stages: (1) cell proliferation for generation of new cells; (2) cell migration which is the movement of newly generated cells to their final destination; and (3) cell differentiation for taking on the appearance and function of a neuron, astrocyte and oligodendrocyte (Zhao et al., 2008). By using BrdU label method, it is shown that the proliferation of NSCs was significantly decreased after ketamine exposure, which is consistent with Dong’s study (Dong et al., 2012). However, there is a discrepancy between our findings and Bai et al. (2013) who revealed that the proliferation of NSCs from human embryonic stem cells (hESCs) was increased followed by 100 μM/L ketamine treatment for 6 h *in vitro*. NSC source and experiment protocol might contribute to the contradiction between our findings and Bai et al.’s (2013).

There are three major cell types from the differentiation of NSCs as neurons, astrocytes, and oligodendrocytes (Wang et al., 2013). It is known that accepting and producing information are basic functions of neurons meanwhile the glia is important for providing the microenvironments to support and nourish the neurons. Hence regulating the NSC maintenance and differentiation determines the growth and function of the developing brain. A better understanding of NSC differentiation is crucial for dissecting the underlying mechanisms associated with ketamine induced injury (Wang et al., 2013). In this study immunostaining was performed with a neuron-specific marker—β-tubulin III antibody and an astrocyte (glia)-specific marker—GFAP antibody which showed that the differentiation of astrocytes was enhanced while the differentiation of neurons was decreased by ketamine exposure, but 50 μM/L MC barely reversed this differentiation changes which may cause potential functional injury. What surprises us is that the effect of MC on differentiation of NSCs was attenuated by LY294002, however, the underlying mechanism need to be exploited in the future.

PI3K is an agonist-activated lipid signaling enzyme that initiate signaling cascades which has emerged as a master regulator of cell through activation of the Akt (Toker, 2000). For instance, low level of NMDA, which has a recognized role in a variety of neuronal physiological and pathological processes, can activate Akt to promote neuronal survival (Sutton and Chandler, 2002). As results from cell viability, immunostaining and western blot strongly suggested that the PI3K signaling pathway is involved in the protective effect of MC in this experiment, we explored the PI3K pathway using LY294002, an inhibitor of PI3K. In agreement with a previous report which showed the activity of Gsk-3β, a substrate of Akt, is suppressed by MC in erythroid progenitors (Somervaille et al., 2001). Our data demonstrated that MC was able to increase the level of Gsk-3β in parallel with the activation of Akt. It was also showed that LY294002 blocked MC-induced phosphorylation of Akt and Gsk-3β. Overall, these results indicating the injury induced by ketamine and the beneficial effect of MC was mediated by PI3K/Akt-dependent Gsk-3β phosphorylation.

In this study, the MC concentrations used ranged from 10 μM to 100 μM in NSCs. This was based on the fact that the neuroprotective dose of MC are approximately at 50–100 μM/L *in vitro* studies (Lin et al., 2001; Wang X. et al., 2003), or probably 200–400 μM/kg *in vivo* tests (Yrjänheikki et al., 1998; Fagan et al., 2004) which is quite different from its antibacterial concentrations (about 4.3–11.0 Mm/L; Zhu et al., 2002; Fagan et al., 2004).

## Conclusion

Our study demonstrated that MC exerted protective effect against the injury induced by ketamine in NSCs and PI3K/Akt/Gsk-3β signaling pathway was involved. The results are helpful in designing clinical studies for treatment of ketamine injury in youngsters. Finally further experiments should be conducted to clarify the safety and underlying mechanism of MC to behave as a neuroprotective drug.

## Author Contributions

YL contributed to the collection, analysis and interpretation of data and drafting of the manuscript. PZ and YL conceived and designed the experiments. YL, SL, NW, PL, WL, JZ and PKG performed the experiments. HL, XC and ZZ revised the article. All authors listed, have made substantial, direct and intellectual contribution to the work, and approved it for publication.

## Funding

This work was supported by the National Natural Science Foundation of China (81071071, 81171247), Key Science and Technology Innovation Team of Shaanxi Province (2014KCT-22) and Science and Technology Development Project of Shaanxi Province grants (2013KTCL03-09).

## Conflict of Interest Statement

The authors declare that the research was conducted in the absence of any commercial or financial relationships that could be construed as a potential conflict of interest.
